# Recent Progress in Antimicrobial Strategies for Resin-Based Restoratives

**DOI:** 10.3390/polym13101590

**Published:** 2021-05-14

**Authors:** Qiannan Sun, Lingyun Zhang, Rushui Bai, Zimeng Zhuang, Yunfan Zhang, Tingting Yu, Liying Peng, Tianyi Xin, Si Chen, Bing Han

**Affiliations:** Department of Orthodontics, Peking University School and Hospital of Stomatology & National Center of Stomatology & National Clinical Research Center for Oral Diseases & National Engineering Laboratory for Digital and Material Technology of Stomatology & Beijing Key Laboratory of Digital Stomatology & Research Center of Engineering and Technology for Computerized Dentistry Ministry of Health & NMPA Key Laboratory for Dental Materials, No.22, Zhongguancun South Avenue, Haidian District, Beijing 100081, China; jelena1023@163.com (Q.S.); changlyht@163.com (L.Z.); bairushui@163.com (R.B.); zhuangzimeng99@163.com (Z.Z.); bdzj_yunfan@163.com (Y.Z.); pengliying@bjmu.edu.cn (L.P.); tinaxin1026@126.com (T.X.)

**Keywords:** antimicrobial, dental materials, dental restorations, polymeric composite

## Abstract

Repairing tooth defects with dental resin composites is currently the most commonly used method due to their tooth-colored esthetics and photocuring properties. However, the higher than desirable failure rate and moderate service life are the biggest challenges the composites currently face. Secondary caries is one of the most common reasons leading to repair failure. Therefore, many attempts have been carried out on the development of a new generation of antimicrobial and therapeutic dental polymer composite materials to inhibit dental caries and prolong the lifespan of restorations. These new antimicrobial materials can inhibit the formation of biofilms, reduce acid production from bacteria and the occurrence of secondary caries. These results are encouraging and open the doors to future clinical studies on the therapeutic value of antimicrobial dental resin-based restoratives. However, antimicrobial resins still face challenges such as biocompatibility, drug resistance and uncontrolled release of antimicrobial agents. In the future, we should focus on the development of more efficient, durable and smart antimicrobial dental resins. This article focuses on the most recent 5 years of research, reviews the current antimicrobial strategies of composite resins, and introduces representative antimicrobial agents and their antimicrobial mechanisms.

## 1. Introduction

Once a tooth defect has formed, restorations are well accepted therapeutic regimens. Because of their tooth-colored esthetics, direct-filling capability and light-curing properties, resin composites are the most commonly used restorative materials and have been increasingly used to replace toxic amalgams [1,2,3]. Typical composite resin is composed of an organic matrix, reinforcing fillers and chemicals that promote or modulate the polymerization reaction [2]. Since its first introduction by Bowen about 50 years ago [4], the application of composites has been extended to various clinical situations, including some that were previously treated only by indirect prosthetic restorations. Extensive improvements mainly focus on the resin matrix and filler systems, mainly to reduce polymerization shrinkage and improve mechanical properties [5,6,7,8].

Although the performance has been greatly improved, the average life of composite restoration is still only around 10 years [9,10]. In the USA, around 60% of all restorations need to be replaced due to restoration failure each year, costing over 5 billion USD per year [11]. Secondary caries is a main reason for composite restorations failure [10,12]. Secondary caries, as with other types of dental caries, is a biofilm-dependent oral disease, resulting in destruction of the tooth structure by acid production from pathogenic bacteria at the tooth–restoration margin. In addition to acid production, enzymes produced by pathogenic bacteria degrade the materials, resulting in leakage at the edge of the restoration–tooth interface, which promotes the formation and progression of caries recurrence [13].

The warm and moist oral environment provides the oral microbiota with rich nutrients and adequate water, making it particularly suitable for microbial growth and proliferation. Bacteria bind to complementary receptors on specific oral surfaces through specific adhesions, and thus colonize different oral surfaces to form biofilms. Restorations of tooth defects cannot prevent the formation of oral biofilms, and it has even been reported that biofilms are more likely to form on the surface of restorations [14]. Therefore, efforts have been devoted to produce innovative resin composites with oral biofilm-suppression properties.

Many researchers have developed novel therapeutic dental composites with antimicrobial and bioactive abilities. In the 1950s, Colton et al. incorporated antibiotic drugs into direct filling resins to impart bactericidal effects to resins [15]. Since then, this strategy has received widespread attention. Many leachable antibacterial agents, such as chlorhexidine (CHX) [16], chitosan [17], silver [18] and nanoparticles (NPs) [19] have been integrated into dental composites. This strategy works by releasing preloaded antimicrobial agents into the oral environment. Another strategy is to covalently anchor the antimicrobial agent to the resin matrix by polymerization. In addition, antifouling dental resins to deter microbial adhesion or adjust the composition of biofilms are also common strategies [20]. In this review, we mainly summarize the articles published predominantly over the past 5 years, and divide the antimicrobial strategies of composite resins into three categories: antimicrobial agent release, contact-dependent strategies and multi-functional strategies. The mechanisms, advantages and disadvantages, as well as the research status of the representative resins in each strategy are summarized. The existing problems of antimicrobial resin are indicated and future prospects are put forward.

## 2. Current Antimicrobial Strategies of Resin Composites

**Figure 1:**
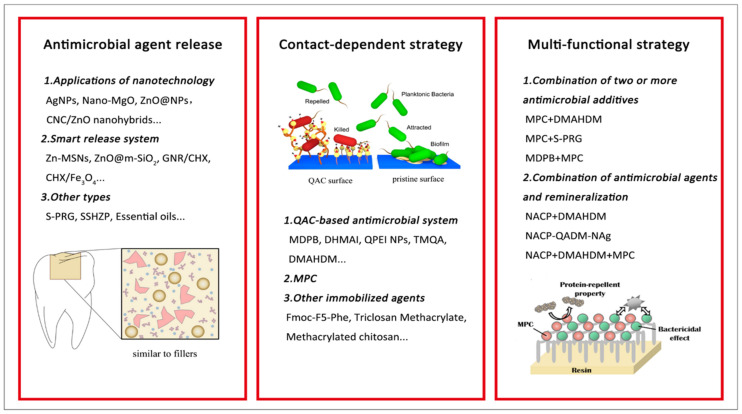
Examples of strategies for material composition modifications toward the design of antibiofilm resin-based composites. AgNPs: silver nanoparticles; MgO: magnesium oxide; CNC/ZnO: cellulose nanocrystal/zinc oxide; Zn-MSNs: zinc-doped mesoporous silica nanoparticles; m-SiO_2_: mesoporous silica; GNR/CHX: Gold nanorods/chlorhexidine; Fe_3_O_4_: ferroferric oxide; S-PRG: pre-reacted glass-ionomer; SSHZP: silver sodium hydrogen zirconium phosphate; QAC: quaternary ammonium compound; MDPB: 12-methacryloyloxy dodecyl pyridinium bromide; DHMAI: quaternary ammonium dimethyl-hexadecyl-methacryloxyethyl-ammonium iodide; QPEI: quaternary ammonium salt polyethyleneimine; TMQA: tetrafunctional methacrylate quaternary ammonium salt monomer; DMAHDM: quaternary ammonium dimethylaminohexadecyl methacrylate; MPC: 2-methacryloyloxyethyl phosphorylcholine; Fmoc-F5-Phe: Fmoc-pentafluoro-l-phenylalanine-OH; NACP: amorphous calcium phosphate. NAg: nano-silver. Adapted, with permission, from [1,21,22].

Due to the significant expense and further loss of dental structure resulting from failed restorations, developing antimicrobial materials to prevent bacterial adhesion and biofilm formation is becoming increasingly important. Considerable efforts have been made to develop dental resins with antimicrobial properties. Three antimicrobial strategies are described in detail below (Figure 1 and Table 1).

### 2.1. Antimicrobial Agent Release

This strategy is that resin composites release the pre-loaded antimicrobial agents into the oral environment over time to kill microorganisms [23]. The advantages of the local release of antibiotics from the resin surface include high local doses of antimicrobial agents at specific sites without exceeding systemic toxicity, thereby minimizing the development of resistance [23]. Leachable antimicrobial agents such as antibiotics, fluoride, silver compounds, chlorhexidine, nano-MgO and other NPs have been widely studied for the possibility of application in composite resins [15,24,25,26,27]. Nevertheless, the release of the antimicrobial agents in these release-based systems is an uncontrolled burst release and it lacks long-term properties. Another limitation of the release system is that with the release of the antibacterial agent, the mechanical properties of the composite resin may be impaired [28,29]. Therefore, recent studies on the release system have mainly been carried out to solve these problems. With the introduction of nanotechnology to either improve existing antibacterial materials or develop new antibacterial fillers, these problems have been resolved to a certain extent [30,31,32].

#### 2.1.1. Applications of Nanotechnology

**Figure 2:**
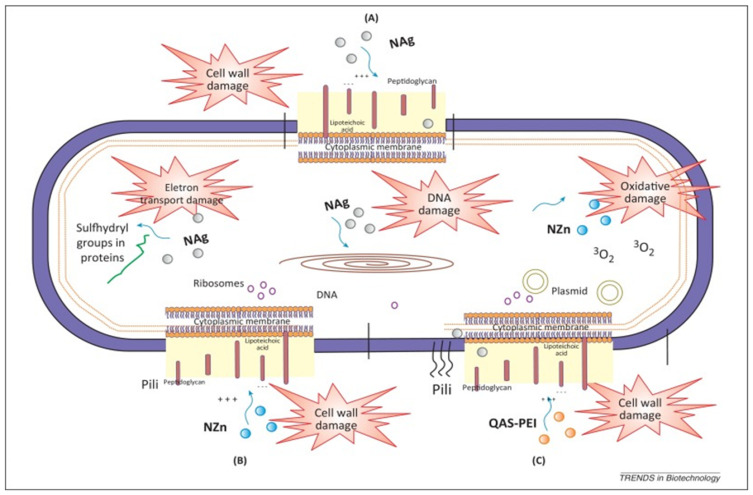
The possible antibacterial mechanisms of different antibacterial agents. (**A**): Schematic representation of antibacterial mechanism of silver nanoparticles (Nag); (**B**): Schematic representation of antibacterial mechanism of zinc oxide nanoparticles (NZn); (**C**): Schematic representation of antibacterial mechanism of quaternary ammonium polyethylenimine (QAS-PEI). Adapted, with permission, from [11].

Nanotechnology is an emerging field of research, with the size of nanoparticles being between 1 and 100 nm [33]. The small size, the high surface area and the capability of releasing high levels of ions at low incorporated amounts make nanoparticles desirable in materials science and biology [33]. The small size of these nanoparticles makes it easier to penetrate the cell membrane, thereby affecting intracellular processes and resulting in higher antibacterial properties [34]. Many nanomaterials, such as Ag, Cu, ZnO and chitosan have been added to composite resin as release additives in order to control biofilms [35]. Among these, silver nanoparticles (AgNPs) have been one of the research hotspots in recent decades [36]. The antimicrobial mechanism of AgNPs has not yet been fully elucidated, but it is generally believed that silver nanoparticles have a bactericidal effect through their own bactericidal effect and release of Ag^+^ [37]. Silver nanoparticles can denature cell membranes and interfere with bacterial signal transduction to cause apoptosis and termination of cell proliferation (Figure 2). In addition, silver ions can destroy the bacterial envelope, interfere with DNA replication and inhibit the synthesis of protein [37]. Most previous reports indicated that adding AgNPs to restoration materials would not affect their mechanical properties, but AgNPs might impair the polymerization process of dental resins [38,39]. Moreover, silver nanoparticles are easy to clump into agglomerates due to their ultrafine size, and silver salts are difficult to dissolve in hydrophobic dental resin monomers, which are challenges faced in AgNP applications [40]. In situ synthesis methods can avoid agglomeration formation during the procedure of mixing AgNPs into resin [41]. Ren et al. showed that AgNPs were successfully synthesized in the resin matrix by reducing silver ions in situ through photoinitiation and proved to have good antibacterial effects [42]. The influence of AgNPs on the color of composite resins and the potential damage to human health limit its scope of use [18,43]. Rodrigues et al. [44] synthesized AgNPs from green tea extract and coated the AgNPs with silica, resulting in light-colored Ag@SiO_2_ nanoparticles. Adding Ag@SiO_2_ NPs to the composite resin can prevent the formation of *Streptococcus mutans* biofilms without compromising esthetics.

Unlike Ag, the ZnO nanoparticles have good safety and a color similar to natural teeth; therefore, they have attracted widespread attention [18]. Firstly, zinc oxide sterilizes through photocatalysis, and when irradiated by ultraviolet or visible light, it produces a series of reactive oxygen species (ROS) to have an antibacterial effect [45] (Figure 2). Secondly, the release of Zn^2+^ from ZnO nanoparticles and the interaction between nanoparticles and bacteria were also supposed to be an important reason for this antibacterial activity [46,47]. Villegas et al. showed that the addition of ZnO nanoparticles to resin composites can simultaneously combat aerobic bacteria and anaerobic bacterial strains [48].

In order not to damage the mechanical properties of the composite resin, Wang et al. prepared cellulose nanocrystal/zinc oxide (CNC/ZnO) nanohybrids through precipitating Zn^2+^ on the surface of CNC and then introducing CNC/ZnO into the dental resin composites. They found that resin composites containing 2 wt% CNC/ZnO nanohybrids possessed higher mechanical properties and showed excellent antibacterial properties with a 78% reduction in bacterial quantity [25].

The nanoparticles could be coated with agents such as phenols and antibiotics, which enhance their performance [34]. Pasha et al. synthesized novel drug-decorated copper nanoparticles using an available drug named Augmentin, which contains amoxicillin [30]. These particles were then added to the resin composite. Experiments showed that they did not have any deleterious effects on the mechanical properties of the composite resin and there was a steady and slow release of copper particles even after 28 days. In addition, some nanocarrier agents for the delivery of drugs, such as montmorillonite and halloysite nanotubes were also used in dental resin composites to achieve controlled release of antibacterial agents [16,31,32].

#### 2.1.2. New Release Systems for Antimicrobial Agents

In order to maintain the mechanical properties of the composite resin and slow down the release rate of the antimicrobial agents, great efforts have been made to improve the original antibacterial filler.

Utilizing drug-carrier particles to store and release drugs without forming voids can bypass these problems and prolong release by the limited diffusion of media into the particle, and subsequent diffusion of the drug out [49]. Mesoporous silica nanoparticles (MSNs) are a promising carrier that can store large amounts of drugs in their pores and extend release by limiting drug diffusion [50]. Bai et al. synthesized Zn-doped mesoporous silica nanoparticles (Zn-MSNs) by a sol–gel method and evaluated their application for dental resin composites. The results showed that the addition of Zn-MSNs to the composite could significantly improve the antibacterial activity, and the antibacterial rate of the composite with 15 wt% of Zn-MSNs reached 100% [51]. One study coated ZnO particles with mesoporous SiO_2_ to fabricate ZnO@m-SiO_2_ [52]. Without reducing the degree of conversion and the depth of cure, the resin composites with bimodal fillers including ZnO@m-SiO_2_ and SiO_2_ NPs have excellent antibacterial properties. However, the current long-term drug release application of MSNs is still affected by the current drug-loading technology and drug release rate [53].

Stewart et al. developed an antimicrobial-drug-silica co-assembled particle system, utilizing octenidine dihydrochloride to form a highly loaded (35% wt.) OCT-silica nanocomposite [54]. They demonstrated that the antimicrobial drug load of drug-templated silica self-assembly-produced particles was significantly higher than conventional drug-loaded mesoporous silica [54]. Delaviz et al. covalently incorporated ciprofloxacin into the backbone of crosslinking divinyl oligomers [55]. This covalent connection is unstable, and the release of the drug is triggered by the degradation of bacteria and saliva on the sensitive bond between the traditional drug antibiotic molecule and the carrier. Zhang et al. developed a polymer–antibiotic conjugate as an antibacterial additive in dental resins [56]. The polymer–drug conjugation linkage should be basically stable in normal healthy biological environments, but susceptible to disease-specific conditions (such as acidic pH, high enzymatic concentrations etc.) to promote drug release when necessary. However, these studies mainly investigated the possibility of the application of these release systems to resin adhesives. There are few studies related to their application in direct-filling resins, which could be carried out in future research.

To maintain a sustained low concentration of fluoride in the oral environment, a material incorporating layered double hydroxides (LDHs) was investigated, which was also rendered fluoride-rechargeable [57,58]. Su et al. developed a novel fluoride-charged LiAl-F LDH and added it to dental composites. The LiAl-F LDHs serve as a fluoride reservoir filler for dental resin composites, with the potential of preventing early-stage carious lesions and secondary caries [57]. A study by Hoxha et al. incorporated MgAl and CaAl LDH into experimental dental composites, and investigated the ability of fluoride release and its effect on the mechanical properties of resin composites. The results showed that CaAl LDH and MgAl LDH composites repeatedly absorbed/released fluoride and maintained the desired physical-mechanical properties [58].

Some smart release systems are also being studied. Chlorhexidine (CHX) is a cationic compound that can bind to the negatively charged walls to destroy cell walls [23]. The controllable and long-term release of chlorhexidine has been widely studied. Boaro et al. developed a composite material with significant antibacterial activity using montmorillonite (MMT), a nanoclay used as a pharmaceutical excipient for controlled drug release, loaded with CHX [16]. Research showed that MMT/CHX can inhibit the growth of *Streptococcus mutans*, *Porphyromonas gingivalis* and *Staphylococcus aureus*, as well as reduce biofilm formation, without compromising mechanical properties. A pH-sensitive stimulus–response system for controlled drug release was developed by Fullriede et al. [59]. In this system, CHX is immobilized in poly(4-vinylpyridine)-modified nanoporous silica nanoparticles. Under physiological pH conditions, the polymer tightly wraps the silica mesopores, preventing the release of chlorhexidine. Under acidic conditions, the molecular chain of polymers is opened due to electrostatic repulsion, so that CHX can be released through the opened pores. Luo et al. proposed a near-infrared (NIR)-mediated chlorhexidine-releasing system. They prepared a novel gold nanorod/chlorhexidine composite, which was responsive to NIR light and had a high drug-loading capacity [60]. Luo et al. also developed a chlorhexidine-releasing system controlled by ultrasound [61]. They synthesized spherical chlorhexidine particles and incorporated them into UDMA-HEMA resins, demonstrating an ultrasonic response and lower CHX release. In addition, Luo et al. [62] also studied the use of a magnetic field to control the release of CHX. Chlorhexidine microspheres containing Fe_3_O_4_ nanoparticles were firstly prepared and then mixed with resin monomers. A magnet was contacted with the resin system to control the distribution of the drug in the material. The Fe_3_O_4_ nanoparticle-functionalized CHX spheres had a magnetic field response characteristic. Therefore, their release kinetics in the dental resin can be controlled by an external magnetic field. These studies provided the possibility of realizing long-term, controlled release of chlorhexidine [60,61,62]. However, the realization of controlled release relies on external stimuli, which may limit its practical application.

#### 2.1.3. New Types of Antimicrobial Agents

Apart from conventionally released antimicrobial agents, researchers have also explored new types of agents to limit bacteria adhesion and biofilm formation. Silver sodium hydrogen zirconium phosphate (SSHZP), a silver-releasing ceramic, was incorporated into light-curing resin composites [63]. In contrast to silver nanoparticles, this submicron-sized antimicrobial material will not cause the typical initial amber or brown discoloration due to the plasma effect.

A surface pre-reacted glass-ionomer (S-PRG) filler, prepared via an acid–base reaction between fluoroboro aluminosilicate glass and a polyacrylic acid, has the capability to release multiple ions such as fluoride (F^−^), borate (BO_3_^3−^), aluminum (Al^3+^), sodium (Na^+^), silicate (SiO3^2−^) and strontium (Sr^2+^) [64,65]. Miki et al. elucidated the mechanism of S-PRG filler’s antibacterial activities and evaluated the association between the release of six ions from a S-PRG filler and the antibacterial activity of the filler [64]. Later, they studied the inhibitory effect of S-PRG fillers on bacterial metabolism [65]. They suggested that the metabolic activities of *Streptococcus mutans* were inhibited in the presence of low concentrations of BO_3_^3−^ and F^−^.

Essential oils contain volatile aromatic components, exerting antibacterial activity against caries-related microorganisms [66]. Composite resin materials modified with essential oils showed antimicrobial properties against *Streptococcus mutans*, *Candida albicans* and *Lactobacillus acidophilus* [67].

### 2.2. Contact-Dependent Strategies

**Figure 3:**
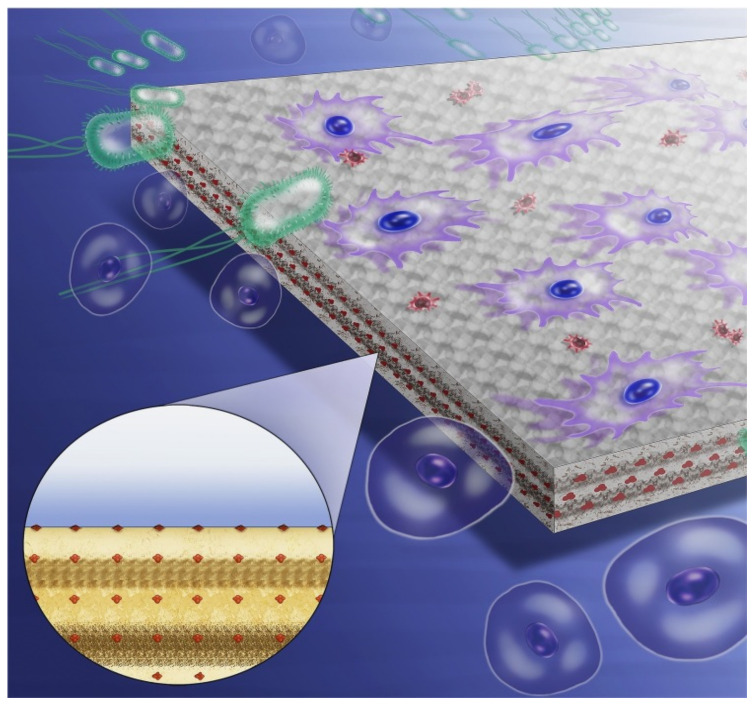
Schematic illustration of the use of contact antimicrobial agents in resin composites. Adapted, with permission, from [69].

To circumvent the uncontrolled burst release of release-based antimicrobial materials, contact-dependent strategies utilize covalent bonds to anchor antibacterial molecules to the polymer backbone [68] (Figure 3). This contact-dependent approach has no adverse effects on the physical and mechanical properties of the loaded materials and can improve and prolong antibacterial activity [14]. Their mode of action includes physical piercing and destruction of bacterial cell walls and membranes as well as viral envelopes [69]. Unlike antibiotics, they will not develop antibiotic resistance [69]. In this strategy, polymers containing insoluble positively charge species such as quaternary ammonium compounds (QACs) have been widely explored [70]. Other polymerizable synthetic and natural compounds such as chitosan [71] and imidazole [72] have also been included. These materials have been proven to effectively reduce the growth of bacteria and microorganisms in a wide range of applications, such as dental materials, medical equipment, food packaging and coatings [21,69,73,74].

However, these contact-dependent antimicrobial restoratives have several limitations: their antibacterial activity may not be as strong as a free-form coating, due to the heavy dependence on the chemical binding process and the direction of the covalent attachment of the antibacterial agent [69]; and dead or compromised bacteria and salivary proteins coating the resin surface may lead to the ineffectiveness of surface killing [20]. The existing problems are introduced in detail below.

#### 2.2.1. Development of QAC-Based Antimicrobial Systems

Although the detailed mechanism of the antibacterial effect of quaternary ammonium compounds has not been determined, it is generally accepted that the interaction between the positively charged (N^+^) sites of a QAC-based resin and the microbial cell wall causes disruption of the integrity of the cell wall [75]. The long lipophilic alkyl chain of quaternary ammonium salt penetrates bacterial cell membranes by anchoring to cell wall components to produce cytoplasmic material leakage, autolysis and bacterial cell death [76].

12-Methacryloyloxy dodecyl pyridinium bromide (MDPB), synthesized by uniting the quaternary ammonium dodecylpyridinium with the methacryloyl group, was the first synthetic quaternary ammonium monomer (QAM) used for antibacterial dental materials, and it has been commercialized as an antibacterial adhesive system (Clearfil Protect Bond, Kuraray Noritake dental Inc., Tokyo, Japan) [14,77]. Subsequently, researchers have conducted extensive investigations into the application of other types of QAMs to antimicrobial dental materials and found that different alkyl chain lengths can cause different antibacterial activities of quaternary ammonium salt resins [70]. As mentioned before, the alkyl chain can penetrate bacterial cells to disrupt membranes like a needle pierces a balloon, so an increase in its length reduces the metabolic activity and acid production of saliva-derived microcosm biofilms. The chain length of 16 dimethylaminohexadecyl methacrylate (DMAHDM) shows the strongest antibacterial ability [78].

Monomethacrylates with only one methacrylate group are the first generation of QAMs and the polymer network of high-concentration monomethacrylate resin blended into the composite resin may inevitably affect its structure and mechanical properties due to the low crosslinking density [23]. To overcome this issue, QAMs with more than two methacrylate groups have been synthesized [79,80,81,82]. He et al. synthesized IPhene, a quaternary ammonium di-methacrylate monomer with an iodine counter-anion. IPhene was then incorporated into bis-GMA/TEGDMA (50/50, wt/wt) with a mass fraction series from 10 wt% to 40 wt%. The results showed that polymers with 20 wt% and 30 wt% IPhene had higher fracture energies than the control polymer, and the samples’ radio-opacity mu increased with an increase in the IPhene mass fraction. However, only composites with 30 wt% and 40 wt% of IPhene showed antibacterial activity [80]. A new quaternary ammonium bis-phenol A glycerolate dimethacrylate (QABGMA) was synthesized and proposed as a microbicidal monomer that has two quaternary ammonium pendant groups. The resin composites incorporated with QABGMA showed good antimicrobial activity against the tested microorganisms. However, resin with ≥10 wt% QABGMA showed obviously reduced viability [83]. Cherchali’s research team discovered that the incorporation of 7.5% quaternary ammonium dimethyl-hexadecyl-methacryloxyethyl-ammonium iodide (DHMAI) in a composite resulted in a strong antibacterial effect, along with acceptable mechanical properties [82]. They then investigated the structural stability and resistance against biodegradation of DHMAI-loaded experimental composites. The results showed that dental composites based on DHMAI possibly enhanced dental composite longevity [79]. Jaymand et al. conducted a series of studies on multi-functional dendritic monomers and found that they can be used to improve the crosslinking degree and performance of resins [84,85,86,87]. Wang et al. synthesized a novel tetrafunctional methacrylate quaternary ammonium salt monomer (TMQA). The incorporation of TMQA in experimental resins can improve the crosslinking degree and displayed antibacterial activity [81].

By harnessing the groundbreaking advancements in nanotechnology and materials science, antibacterial solid nanoparticles (NPs) with QAS functionality have been proposed. Quaternary ammonium salt polyethyleneimine (QPEI) nanoparticles are a contact-active bactericide when mixed with various resin-based materials [88,89,90]. Pietrokovski et al. evaluated the antibacterial activity against *Streptococcus mutans* and *Actinomyces viscosus* of materials incorporating QPEI nanoparticles. Moreover, the influence of polishing and smoothing the surface of dental restorations on the antibacterial effect was examined. The results showed that foundation material incorporating 1% wt/wt QPEI nanoparticles exhibited strong antibacterial activity against *Streptococcus mutans* and *Actinomyces viscosus*s. Polishing the surface of the material did not affect its antibacterial activity [91]. Zaltsman et al. optimized the process of synthesizing QPEI nanoparticles by controlling the addition of NaHCO_3_ to enhance its antibacterial activity [89].

#### 2.2.2. Introduction of 2-Methacryloyloxyethyl Phosphorylcholine (MPC)

**Figure 4:**
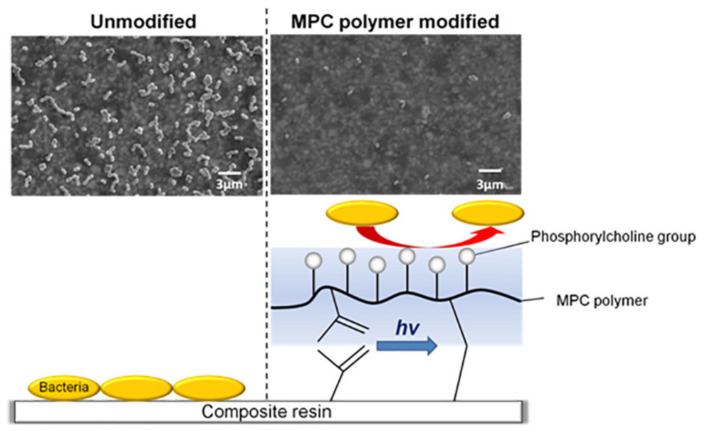
Schematic illustration of the use of 2-methacryloyloxyethyl phosphorylcholine (MPC) in resin composites. Adapted, with permission, from [93].

To prevent salivary protein coating on the resin surface from interfering with the action of non-releasable antimicrobial agents, protein-repellent agents have been introduced [92,93]. Earlier, Muller et al. discovered that poly(ethylene glycol) and methacrylate monomers with pyridinium groups were immobilized to produce protein-repellent functions [92]. Combining protein- and bacteria-repellent agents with the bactericidal function of charged cationic groups is promising [92]. MPC, a methacrylate with a phospholipid polar group in the side chain, is a hot spot of recent research. Since bound water can cause protein adsorption, the large amount of free water around the phosphorylcholine groups of MPC polymers is considered to be effective in preventing protein adhesion [94] (Figure 4).

Koyama et al. synthesized a MPC polymer that could bind on the surfaces of the resin composite in situ. The modified surface showed obvious resistance to oral protein adsorption and bacterial adhesion, even when the surface was brushed with a toothbrush [93]. Fujiwara et al. conducted a single-blind crossover clinical trial to evaluate the effect of 5% MPC polymer mouthwash after 5 h on oral microflora. They suggested that an MPC polymer coating in the oral cavity may suppress oral bacterial adherence [95]. Recent research has focused on the combined application of MPC and other antimicrobial agents, such as DMAHDM [96], S-PRG [97] and MDPB [22]. The relevant content will be explained in detail in the multi-functional strategy module below.

#### 2.2.3. Development of Other Immobilized Antimicrobial Agents

There have been many attempts to develop new bio-stable and antidegradation immobilized antimicrobial agents [98,99,100,101]. The nanoassemblies formed by Fmoc-pentafluoro-l-phenylalanine-OH (Fmoc-F5-Phe) have been functionally incorporated within resin-based composites. These nanoassemblies, comprising both functional and structural subparts, have excellent antibacterial capabilities and remineralization enhancement capabilities. Incorporated with the 2 w/w% nanostructures formed by Fmoc-F5-Phe, composite restoratives retain both the biocompatibility and the mechanical strength of the original restorative [98].

Several studies have modified antimicrobial agents, for example, by connecting them to a methacrylate monomer, so that they can be added to the crosslinked network of the resin composite [99,100]. A triclosan methacrylate (TM) monomer was developed and incorporated into an experimental resin composite. The TM-containing composite has harmful effects at the molecular and cellular levels on *Streptococcus mutans*, causing a reduction in the virulence of these microorganisms [99]. Stenhagen et al. prepared methacrylated chitosan (CH-MA) for incorporation into dental composites. While it reduced biofilm formation on resin-based composites’ surfaces, it posed challenges to the mechanical properties of the resin [100]. Because of the rapid release of silver ions and possible biological toxicity, Srivastava and Sun covalently immobilized silver sulfadiazine onto glass fillers and used them in dental restorative formulas. The modified composites provided non-leachable and contact-dependent antimicrobial effects [102]. Sulfadiazine promotes the formation of a complex of silver and the DNA of bacteria [103], which ultimately leads to the death of the bacteria, showing a synergistic effect with silver.

### 2.3. Multi-Functional Strategies

Strategies involving antimicrobial agent release and contact killing have been developed for many years. However, these two strategies have not achieved complete success due to their inherent shortcomings such as burst release and weak antimicrobial ability [23]. Modified resin composites are expected to play a better role in the complex and diverse oral environment through a possible synergistic effect of the combination of various strategies. The multi-functional strategy can be generally divided into two categories: a combination of two or more antimicrobial additives, and a combination of released/non-leachable agents and remineralization. The combination of antibiofilm agents and remineralization strategies is the frontier of the research. Here, several selected examples will be introduced (Table 2).

Efforts have been made to combine MPC with antimicrobial agents to gain bacteria-eradicating properties and great protein-repellent properties. Zhang et al. incorporated both MPC and DMAHDM into a resin composite for the first time in 2015. They observed that the composite containing MPC and DMAHDM possessed the double benefits of protein-repellent and antibacterial capabilities [111]. After that, a study of the effects of water-aging for 180 days on the protein resistance and bacteria-killing ability of a composite containing MPC and DMAHDM was conducted [104]. Lee et al. confirmed the synergetic effect between a S-PRG filler and MPC. The addition of 3% MPC to resin composite containing S-PRG inhibited multi-species biofilm formation, while maintaining or even enhancing the inherent properties [97].

Composites containing nanoparticles of amorphous calcium phosphate (NACP) strongly release calcium (Ca) and phosphate (P) ions at an acidic pH, which can intelligently start the remineralization process [112]. Antimicrobial agents and NACP have been combined in dental composites, so that while the antibacterial agent reduces the biofilm, Ca and P ions remineralize lesions and inhibit the formation of dental caries [105,113]. Resin composites incorporated with both NACP and DMAHDM showed antibacterial, remineralization and lower shrinkage stress properties [106]. Balhaddad et al. explored the optimization and formulation of dental composites with the addition of DMAHDM and NACP. The mechanical properties were not decreased, and the microbiological assays were substantially reduced in the presence of 5% DMAHDM [107]. Recently, composites containing leachable calcium phosphate (CaP) that could be recharged to provide long-term antibacterial functions have been developed [114]. Studies on the modification of resin composites by incorporating rechargeable NACP and DMAHDM have been carried out. All these studies have shown that the novel rechargeable NACP and DMAHDM modified resin composites possess long-term remineralization and antibacterial properties, and hence the potential to inhibit caries [108,109,110].

Although numerous antimicrobial agents have been developed and have shown general effectiveness against microorganisms, different biocidal approaches have different killing mechanisms, and each method is effective only for a specific type of bacteria. Under this premise, the single killing mechanism becomes less effective, and the multi-functional strategy is more promising [115].

## 3. Challenges Faced by Antimicrobial Dental Restorations

### 3.1. Biocompatibility

The rapid development of novel materials in dental applications makes the public more and more aware of the biological risks and limitations of these materials. The biocompatibility of biomaterials used to replace or fill biological tissues such as teeth has already aroused a high concern in patient health care [116].

Studies believed that both AgNPs themselves and Ag^+^ released by nanoparticles are toxic, promoting membrane damage, protein oxidative denaturation, mitochondrial dysfunction and DNA damage, and leading to apoptosis [117,118]. Sudhakaran et al. discovered that ZnO nanoparticles induced anomalies in the histology, ion content and antioxidant system within the liver [119]. The potential toxicity of releasable agents brings challenges for their usage. Therefore, in the process of using the released antimicrobial agents to modify dental resin composites, special attention should be paid to the release kinetics to avoid excessive accumulation in the human body and cause non-reversible harm to the human body [118,119].

Immobilized antibacterial agents will not release antibacterial ingredients into the environment and several studies have shown their good biocompatibility [82,84,87,88]. However, the effect of biodegradation on many antimicrobial monomers is unknown. For those which contain unprotected and easily hydrolyzed bonds, degradation may happen and thus release toxic degradation products, even though they are covalently attached to the matrix [49]. In sum, the long-term biocompatibility of antimicrobial modified resin composites should be studied.

### 3.2. Drug Resistance

Recently, a study investigated the drug resistance of antimicrobial agents against *Streptococcus mutans*, *Streptococcus sanguinis* and *Streptococcus gordonii*, and evaluated biofilms on resins with repeated exposure for 20 passages. The results revealed that DMAHDM had no drug resistance, and DMAHDM resin reduced biofilm CFU by 3–4 log, even after exposure for 20 passages. However, *Streptococcus gordonii* developed a moderate drug resistance against DMADDM and CHX. [120]. Other studies also concluded that QAC-based agents had no drug resistance [121,122]. The similarity among these reports is that they only investigated planktonic bacteria instead of biofilms. Orazi and O’Toole reported that bacteria embedded in a biofilm are increasingly recognized as having greatly enhanced resistance to many antibiotics relative to their planktonic counterparts [123]. The research on drug resistance should not be limited to the impact on a single species. It is necessary to simulate the oral environment as much as possible and conduct research under the conditions of a biofilm formed by multiple species.

### 3.3. Controlled Release of Antimicrobial Agents

As mentioned above, many related research has been carried out to achieve the controlled release of antimicrobial agents under the premise of maintaining the mechanical properties [49,50,51]. Research focuses have included the development of drug-carrying systems [52,54], smart release systems [60,62] and recharging materials [57,58]. However, the antimicrobial agents contained in the drug-carrying systems and the smart release systems are not endless. Once the antimicrobial agents have been completely released, the composite will lose its antimicrobial effect [23]. For rechargeable materials, the charging process needs to be repeated, increasing the treatment procedures [57,58]. The development of a controlled release system that would not become exhausted is a major challenge we face.

### 3.4. Methods of Assessing Antimicrobial Properties

Once a new antimicrobial resin composite has been developed, a series of assessment will be carried out to evaluate whether it holds the promise of inhibiting bacterial adhesion and reducing biofilm formation. Several methods are available to determine bacterial viability, including the gold standard, colony-forming unit [20] determination, followed by the evaluation of biofilms via microscopy. The other methods include metabolic activity evaluation, inhibition zone testing via disc diffusion and lactic acid production, and measurement of turbidity via spectrophotometry [124]. However, one method that solely relies on metabolic activity or membrane integrity might yield misleading results [20]. In addition, 54% of the tests used single-species biofilms of *Streptococcus mutans*, and only 1% used biofilms with more than three species [124]. The fundamental differences between single- and multi-species biofilms could easily lead to two completely different results for the same material [125]. Because of these limitations, the creation of a more standardized testing technique to better evaluate the effectiveness of antimicrobial materials is called for.

## 4. Conclusions

To prevent high failure rates and prolong service life, it is effective to arm the composite resin with antimicrobial agents. Though various strategies have been used to modify dental composite resins, few antimicrobial agents have been used clinically due to their inherent defects. In the future, we should focus on the development of smart, longer lasting and biosafe agents, while maintaining or improving the mechanical and curing properties of the composite resin itself. In the meantime, a standardized testing process needs to be established to accurately evaluate the effectiveness and biosafety of the antimicrobial composite resin in inhibiting the occurrence of dental caries, as well as comparing the antimicrobial properties of different restorative materials.

## Figures and Tables

**Table 1:** Advantages and disadvantages of three antibacterial strategies.

Name	Advantages	Disadvantages
Antimicrobial agent release	High local doses of antimicrobial agents at specific sites, less systemic toxicity	Short-acting and compromised mechanical properties
Contact-dependent strategy	No adverse effects on the physical and mechanical properties of the loaded materials, improved and prolonged antibacterial activity	Relatively weak antimicrobial activity and surface biofouling
Multi-functional strategy	Synergistic antibacterial activity	Selection of more effective combinations

**Table 2:** Summary of synergistic antimicrobial combinations.

Speciation	Types of Dental Composite	Microorganisms Tested	Test Method for Antimicrobial Activity	Results	Reference
MPC, DMAHDM	BisGMA, TEGDMA	Human saliva	CFU counts; live/dead assay; MTT assay; BCA approach	Strongly deterred protein adhesion and diminished biofilm viability	[104]
NACP, QADM, NAg	BisGMA, TEGDMA	Human saliva	Live/dead staining; MTT assay; lactate analysis; CFU counts	Significantly stronger antibacterial capability than using QADM or NAg alone	[105]
NACP, DMAHDM	UDMA, TEG-DVBE	Human saliva	Live/dead staining assay; CFU counts; lactate dehydrogenase enzymatic method; CV staining	Demonstrated long-term antibacterial activity.	[106]
DMAHDM, NACP	BisGMA, TEGDMA	Human saliva	Live/dead staining; MTT assay; enzymatic method; CFU counts	All the microbiological assays were substantially reduced in the presence of 5%DMAHDM	[107]
NACP, DMAHDM	EBPADMA, PMGDM	Human saliva	Live/dead staining; MTT assay; enzymatic method; CFU counts	NACP-DMAHM inhibited biofilms’ metabolic activity and lactic acid, and reduced biofilm colony-forming units (CFU) by 3–4 log	[108]
NACP, DMAHDM, MPC	EBPADMA, PMGDM	Human saliva	BCA method; live/dead staining; MTT assay; CFU counts	3% MPC+3% DMAHDM inhibited biofilm growth and viability, reducing biofilm CFU by 3 log	[109]
NACP, DMAHDM	EBPADMA, PMGDM	*Streptococcus mutans*	Live/dead staining assay; CFU counts; lactate dehydrogenase approach	NACP-DMAHM composite reduced biofilm acid, and reduced CFU by 4 log	[110]

Abbreviations: BisGMA: bisphenol glycidyl dimethacrylate; TEGDMA: triethylene glycol dimethacrylate; CFU: colony-forming units; QADM: quaternary ammonium dimethacrylate; BCA: bicinchoninic acid; UDMA: urethane dimethacrylate; TEG-DVBE: triethylene glycol divinylbenzyl ether; CV: crystal violet; EBPADMA: ethoxylated bisphenol A dimethacrylate; PMGDM: pyromellitic glycerol dimethacrylate.

## Data Availability

The data presented in this study are available on request from the corresponding author.
